# Identification of a novel deletion within *ALDH3A2 gene in an Iranian Family* with Sjögren–Larsson Syndrome

**DOI:** 10.1002/ccr3.1235

**Published:** 2017-11-22

**Authors:** Maryam Taghdiri, Atie Kashef, Majid Fardaei, Mohammad Miryounesi

**Affiliations:** ^1^ Genetic counseling Center Welfare Organization Shiraz Iran; ^2^ Comprehensive Medical Genetics Center Shiraz University of Medical Sciences Shiraz Iran; ^3^ Department of medical Genetics Shiraz University of Medical Sciences Shiraz Iran; ^4^ Genomics Research Center Shahid Beheshti University of Medical Sciences Tehran Iran

**Keywords:** *ALDH3A2* gene, congenital neuroichthyosis, deletion, Sjögren–Larsson syndrome

## Abstract

Sjögren–Larsson syndrome (SLS) is a rare type of congenital ichthyosis with neurological problems and intellectual disability. Homozygous mutations in *ALDH3A2 gene* are known to be responsible for this syndrome. Here, we report an Iranian family with congenital SLS bearing a novel two‐base‐pair deletion within *ALDH3A2* genomic sequence. Our finding expands the mutation spectrum of *ALDH3A2* that is applicable for further molecular studies and management of SLS.

## Background

Sjögren–Larsson syndrome (SLS) is an autosomal recessive neuroichthyosis caused by mutations in the aldehyde dehydrogenase family 3 member A2 (*ALDH3A2*) gene. *ALDH3A2* encodes a fatty aldehyde dehydrogenase (FALDH) 1. The major characteristics of this metabolic disorder include ichthyosis, mental retardation, spasticity, and seizure.

To date, more than 90 different mutations of *ALDH3A2* are published in patients with SLS 2. Almost all small and large deleterious deletions of ALDH3A2 reported so far result in the major symptoms of FALDH deficiency and SLS 3, 4. This is a genetic condition in which the specific membrane‐bound FALDH is deficient due to a lack of normal alleles of *ALDH3A2*. This enzyme is responsible for converting long‐chain fatty aldehydes into their corresponding fatty acids and detoxifying‐free aldehydes from different lipid degrading pathways. In the absence of FALDH, toxic metabolites cause symptoms in various tissues 5. In skin, accumulation of fatty alcohol in keratinocytes stimulates synthesis of wax esters and alkyl‐2‐acyl‐glycerol that transform the intercellular membranes in the stratum corneum and result in abnormal permeability to water. Consequently, hypertrophic stratum corneum phenotype will be manifested as ichthyosis 6.

## Methods

### Clinical reports

**Figure 1:**
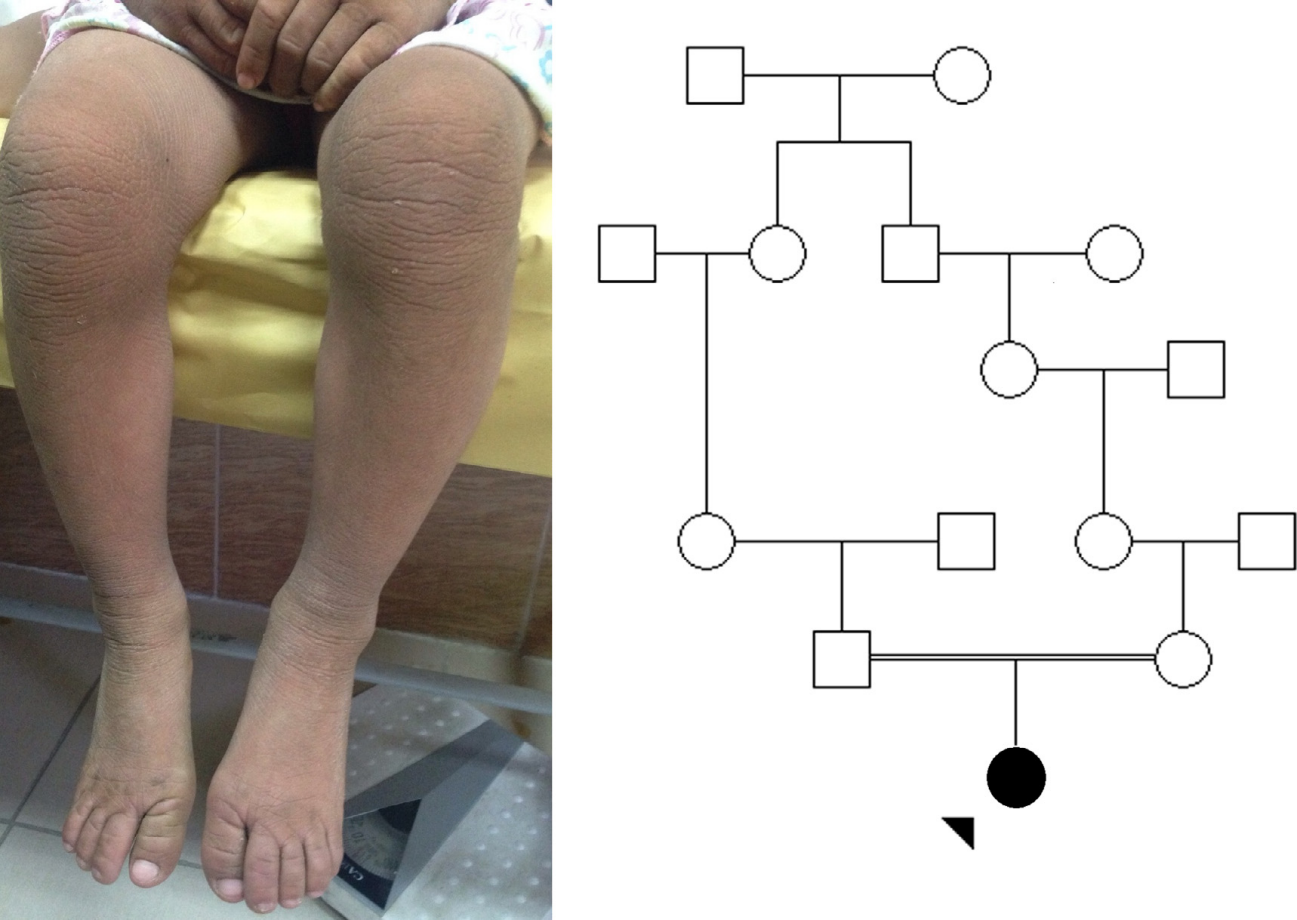
The cutaneous exhibition of SLS in proband (left); dry, rough, and scaly skin of hands, both legs and feet. She exhibits generally dispersed ichthyosis, pruritus, and hyperkeratosis. The pedigree of the studied family indicates consanguineous marriage and autosomal recessive pattern of inheritance (right).

Here, we report a 4‐year‐old girl with SLS symptoms from an Iranian family with consanguineous marriage. She mainly suffers from dry, rough and scaly skin, and walking disability. She exhibits generally dispersed ichthyosis over the whole body, pruritus, and hyperkeratosis (Fig. 1).

Her parents claimed that pregnancy and delivery has been uneventful, and she had no collodion membrane at birth. The progressive ichthyosis has been manifested with slight scaling in the first week after birth. To date, she has not been able to stand and walk on her own. The lower spastic muscles lead to scissoring gait and hyperactive deep tendon reflex (DTR). She has had seizure in 15 months old and 2 years old confirmed with abnormal Electroencephalography (EEG) results that is controlled with medication. EEG shows slow background activity with abnormal sleep EEG due to the presence of some dysrythmic discharges in both hemispheres. Brain MRI reveals general change in signal at periventricular and centrum semiovale white mater which is a clue of dysmyelination. The result of funduscopic examination is normal. The external ear formation and audiogram are normal. She clearly exhibits learning problems and delayed speech.

### Genetic testing

We obtained the consent form from the parents for blood sampling, according to the guidelines of local ethic committee of Welfare Organization.

Genomic DNA was extracted from peripheral blood samples. Targeted mutation analysis was performed using standard sequencing. We performed presequencing PCR amplification for all *ALDH3A2* exons and exon–intron boundaries. Specific primers were designed by AllelID 7.5v software, PREMIER Biosoft International,3786 Corina Way, Palo Alto CA 94303‐4504, USA. Purified PCR products were directly sequenced using standard Sanger method, and sequence reads were aligned and analyzed using DNASTAR software,1600 Tysons Blvd, Suite 800, McLean, VA 22102, USA.

## Results

**Figure 2:**
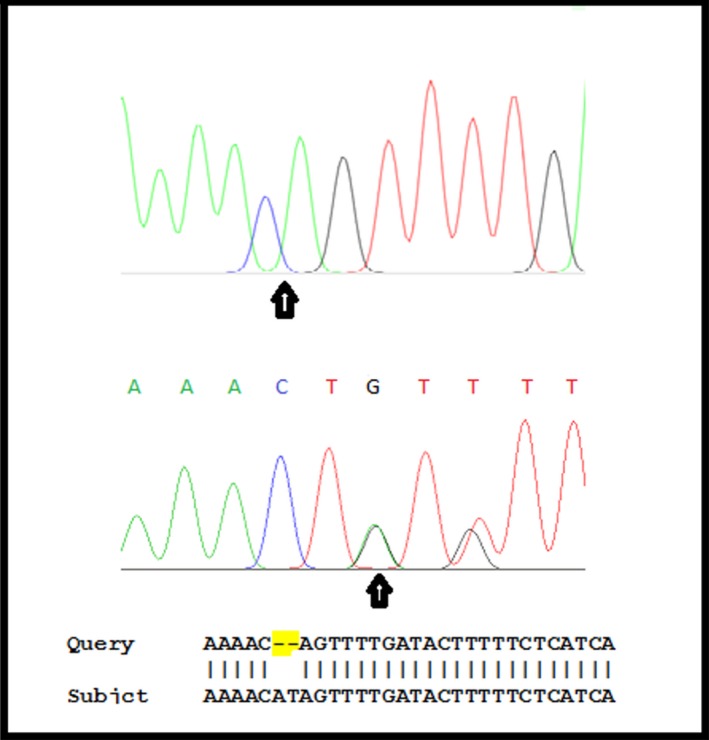
Partial sequence chromatogram (upper) showing novel two‐bp deletion in the ninth exon of ALDH3A2 gene in proband (Homozygote c.1241_1242delAT), in comparison to the chromatogram for heterozygous parents (lower). Sequence alignment for deleted region, proband sequence is aligned against normal sequence.

Sequencing analysis of *ALDH3A2* gene in proband DNA revealed a novel two‐bp homozygous deletion, c.1241_1242delAT, resulting in a frame shift and protein truncation (p.His414Gln*fs3). Heterozygosity for this deletion was also confirmed in parents using sequencing analysis (Fig. 2).

## Discussion

SLS diagnosis should be considered in every case of congenital ichthyosis accompanied by neurological features 7. Main dermatologic problems of SLS in early infancy include erythemas that consequently turn to dry, rough, and scaly skin with brownish or yellowish tone. Differential diagnosis should be considered between other types of congenital ichtyosis including Ichthyosis vulgaris (MIM #146700), X‐linked ichthyosis (MIM #308100), Lamellar ichthyosis (MIM #242300), Congenital ichthyosiform erythroderma (MIM #242100), and Epidermolytic hyperkeratosis (MIM #113800). When neurologic symptoms are present, neuroichthyotic disorders must be considered including Pseudo‐SLS, Refsum disease (MIM #266500), Rud syndrome (MIM 308200), Chanarin–Dorfman syndrome (MIM #275630), Gaucher disease type 2 (MIM #230900), and Multiple sulfatase deficiency (MIM #272200) 8, 9.

In the lack of access to a routine diagnosis test for FALDH or FAO activity in cell culture, histochemical staining for FAO activity in a fresh skin biopsy is an alternative method to confirm FALDH deficiency. Eventually, the affirmative diagnosis will be obtained by DNA sequencing and identifying mutations in the *ALDH3A2* gene. The human *ALDH3A2* gene is 31 kb long and consists of 11 exons that are numbered 1–10 with an alternative exon (exon 9′) situated between exons 9 and 10 10, 11. This gene encodes FALDH, a protein which consists of 485 amino acids. FALDH protein structure consists of a carboxy‐terminal domain which plays a vital role in microsomal membrane anchoring. Deletions are the most frequently reported mutations in *ALDH3A2* gene 4. We report a novel two‐bp pair deletion within exon 9 of this gene, and the pathogenic significance of this mutation is proved by the major features of SLS phenotype in our patient. This deletion results in a frame shift within the coding sequence which is translated into a stop codon by three amino acids after Glutamine 414 of amino acid sequence (p.His414Gln*fs3). The domain structure of FALDH protein includes a NAD‐binding domain (residues 1–79 and 103–208), a catalytic domain (residues 209–419) and a C‐terminal oligomerization domain (residues 82–102 and 420–443). The C‐terminal residues 445–460 construct an alpha helix which is not present in any other forms of soluble ALDHs 5. Further molecular experiments are necessary to reveal the exact pathogenic function of this truncated protein.

Previously, a Turkish family was reported with two siblings, both presenting congenital ichthyosis. A homozygous four‐bp deletion (1384–1387delGAAA) in exon 9 of the FALDH gene was detected in both patients, which results in premature termination of translation at codon 474. Deleted amino acids are supposed to code the transmembrane domain (amino acids 464–480) and membrane retention signal. Hence, truncated FALDH cannot be anchored to the microsomal membrane. The elder sister showed neurologic symptoms including spastic diplegia of the legs, mental retardation and glistening white dots of the retina, but the younger brother only showed subtle neurologic signs but no retinal abnormality 12. Two other deletions in exon 9 (1291–1292delAA and 1297–1298delGA) have been reported in Japanese and European families. These mutations cause frame shifts that produce truncated FALDH protein and SLS phenotype 11. The other deletion (c.1223delG) within exon 9 is known to be responsible for translation stop at codon 427 and truncated FALDH protein. The 15 months patient was born with collodion, shows ichthyosis, mild mental retardation, and nonambulatory spastic diplegia 13.

Based on our clinical findings, we used the standard Sanger sequencing as a gene‐targeted mutation detection approach. However, this method cannot detect large contiguous gene deletions described in few homozygote and compound heterozygote cases of SLS. Large deletions on 17p11.2 chromosome site encompassing *ALDH3A2* has been detected using long‐distance inverse PCR and microarray‐based comparative genomic hybridization and are considered to account for about 5% of mutant alleles in SLS 3.

The patient we studied suffers from the major signs of SLS including ichthyosis with hyperkeratosis and pruritus, intellectual disability, and spastic diplegia. However, the ocular defects and preterm delivery which is mentioned in some other reports of SLS were not detected. Although ocular symptoms were not found in this patient probably because of her young age (glistening white dots and photophobia usually appear after 4 years old), in the other few reports of SLS in Iran, these two signs were not also detected 14, 15. So far, systematic genotype–phenotype correlations in SLS have not been available due to remarkable heterogeneity and the broad distribution of most mutations throughout patients from small families 13. We hope these valuable correlations become more feasible when more patients with SLS are diagnosed with the same genotypes.

Our attempt on mutation analysis for patients with SLS will bring the knowledge that is applicable in different facets; in a population‐based approach, we aim to build up a comprehensive panel of mutations for this disorder which will considerably decrease the costs and complication of medical genetic counseling and preimplantation/prenatal genetic tests. Moreover, mutations data will supply further basic researches on pathomechanisms of this disease in order to improve the efficacy of medical treatments.

## Authorship

MT: involved in clinical evaluation and diagnosis, writing the clinical report. AK: provided scientific support for writing, editing and documentation. MF: involved in genetic testing and technical support. MM: involved in clinical evaluation and diagnosis.
